# Analysis of the miRNA expression from the adipose tissue surrounding the adrenal neoplasia

**DOI:** 10.3389/fcvm.2022.930959

**Published:** 2022-07-28

**Authors:** Antonio Concistrè, Luigi Petramala, Francesco Circosta, Priscilla Romagnoli, Maurizio Soldini, Marco Bucci, Domenico De Cesare, Giuseppe Cavallaro, Giorgio De Toma, Francesco Cipollone, Claudio Letizia

**Affiliations:** ^1^Department of Clinical, Internal Medicine, Anesthesiology and Cardiovascular Sciences, “Sapienza” University of Rome, Rome, Italy; ^2^Department of Translational and Precision Medicine, “Sapienza” University of Rome, Rome, Italy; ^3^Department of Medicine and Aging Sciences, University “Gabriele d'Annunzio” of Chieti-Pescara, Chieti, Italy; ^4^Department of Surgery “Pietro Valdoni, ” “Sapienza” University of Rome, Rome, Italy

**Keywords:** miRNAs, primary aldosteronism, aldosterone-secreting adrenal adenoma, adipose tissue, adrenalectomy

## Abstract

**Background:**

Primary aldosteronism (PA) is characterized by several metabolic changes such as insulin resistance, metabolic syndrome, and adipose tissue (AT) inflammation. Mi(cro)RNAs (miRNAs) are a class of non-coding small RNA molecules known to be critical regulators in several cellular processes associated with AT dysfunction. The aim of this study was to evaluate the expression of some miRNAs in visceral and subcutaneous AT in patients undergoing adrenalectomy for aldosterone-secreting adrenal adenoma (APA) compared to the samples of AT obtained in patients undergoing adrenalectomy for non-functioning adrenal mass (NFA).

**Methods:**

The quantitative expression of selected miRNA using real-time PCR was analyzed in surrounding adrenal neoplasia, peri-renal, and subcutaneous AT samples of 16 patients with adrenalectomy (11 patients with APA and 5 patients with NFA).

**Results:**

Real-time PCR cycles for miRNA-132, miRNA-143, and miRNA-221 in fat surrounding adrenal neoplasia and in peri-adrenal AT were significantly higher in APA than in patients with NFA. Unlike patients with NFA, miRNA-132, miRNA-143, miRNA-221, and miRNA-26b were less expressed in surrounding adrenal neoplasia AT compared to subcutaneous AT in patients with APA.

**Conclusion:**

This study, conducted on tissue expression of miRNAs, highlights the possible pathophysiological role of some miRNAs in determining the metabolic alterations in patients with PA.

## Introduction

In addition to moderate–severe hypertension or resistant hypertension (RH), primary aldosteronism (PA) is a frequent cause of secondary hypertension (up to 10% in newly diagnosed arterial hypertension), associated with important target organ damage and several complications related to pressure overload and pleiotropic effects of aldosterone on the cardiovascular system (1, 2).

Primary aldosteronism is characterized by several metabolic changes, such as metabolic syndrome (3), myocardial hypertrophy and fibrosis (4), atrial fibrillation (5), and in particular, heart failure (HF) and deleterious effects on vascular remodeling secondary to endothelial dysfunction (6–8).

Adipose tissue (AT) represents a complex and active metabolic and endocrine organ, taking part in all processes characterized by insulin resistance and obesity-related diseases. Interestingly, visceral AT seems to have significantly greater metabolic activity than the subcutaneous site that is able to produce several molecules biologically active (adipokines and proinflammatory and procoagulant cytokines), expressing receptors for several molecules (9). Previously, we found that the visceral AT altered the balance between several adipokines (leptin, resistin, and adiponectin) in patients with PA compared to patients with essential hypertension (EH) (3), suggesting a possible mechanism of impaired metabolism in patients with PA.

Mi(cro)RNAs (miRNAs) are a class of non-coding small RNA molecules that regulate gene expression by targeting miRNAs, triggering translational repression, or triggering RNA degradation, representing critical regulators in several cellular processes (differentiation, proliferation, and apoptosis) (10). Thus, miRNAs have been assessed as crucial elements in significant pathological processes, including various types of cancer, cardiovascular diseases, infectious, inflammatory, and autoimmune conditions, diabetes mellitus, and obesity-related diseases (11, 12). Concerning the latter, miRNAs seems to be involved on the development of obesity, metabolic syndrome, and insulin resistance (13).

The aim of this study was to evaluate the behavior of the tissue expression of some miRNAs at the visceral and subcutaneous AT in patients undergoing adrenalectomy for aldosterone-secreting adrenal adenoma (APA), compared to samples of AT obtained in patients undergoing adrenalectomy for non-functioning adrenal mass (NFA). The selection of miRNAs was based on data reported in previous studies, in particular we selected miRNAs previously evaluated in literature because they are involved in metabolic and inflammatory regulation of AT (14).

## Subject and methods

### Patients

From January 2019 to December 2020, 16 consecutive patients were enrolled in our Tertiary Center of Secondary Arterial Hypertension: 11 patients with APA and 5 patients with NFA. The diagnosis of PA was performed according to the Endocrine Society Guidelines (2). NFA was diagnosed in patients with adrenal incidentalomas and normal adrenal hormone secretion. Surgical resection was done if the NFA was larger than 6 cm or if the lesion enlarged by more than 20% (in addition to at least a 5-mm increase in maximum diameter) during a follow-up period of 12 months as suggested by the European Guidelines (15). Patients with confirmed biochemical and/or histopathological diagnoses of APA or NFA were included. Plasmatic cortisol, 24-h urinary cortisol, and 24-h urinary metanephrines were measured, and patients with cortisol and catecholamines overproduction were excluded from the study. Subjects with coronary artery disease (CAD), HF, valvular heart disease, pericardial disease, cerebrovascular disease, peripheral arterial disease, kidney or liver disease, or with a history of cerebrovascular or cardiovascular events were also excluded. Patients taking lipid-lowering drugs, hypoglycemic drugs, or corticosteroids were also excluded. All the study participants followed a regular dietary habit with a salt intake of 140 mEq/day and a potassium intake of 50–75 mEq/day. All patients had stable body weight for 6 months before the study. In order to determine hypertension-related organ damage, we recorded a transthoracic 2D-guided M-mode echocardiogram using commercially available equipment (Esaote MyLab Omega). Standard parasternal and apical views were obtained with patients lying in the left lateral decubitus position. Left ventricular mass (LVM) and LVM indexed for body surface area (LVMi) were calculated according to the American Society of Echocardiography (ASE) guidelines (16). A 24-h ambulatory blood pressure monitoring (ABPM) was performed using the oscillometric technique, which involves a portable lightweight, non-invasive monitor with a self-insufflating cuff (Spacelabs Medical, 90207, Issequah, WA, USA). ABPM readings were obtained at 15-min intervals from 6 A.M. to midnight and at 30-min intervals from midnight to 6 A.M. All patients underwent laparoscopic adrenalectomy by the same operator. The postoperative course was uneventful for all patients. During surgery, a small fragment (<5 mm) of AT was obtained from 2 different depots for each site: (a) surrounding adrenal neoplasia, (b) peri-renal, and (c) subcutaneous depots. Real-time quantitative polymerase chain reaction (PCR) for miR-132, miR-143, miR-221, miR-26a, and miR-26b was performed. The selection of miRNAs was based on data reported in previous studies (14, 17–24). After 1-year follow-up after adrenalectomy (median follow-up 12.1 ± 2.1 months), we collected data on anthropometric parameters and physical examination, pharmacological treatment, blood samples, and 24-h ABPM. All methods were performed in accordance with relevant guidelines and regulations. All subjects gave their informed consent for inclusion before they participated in the study. The study was conducted in accordance with the Declaration of Helsinki, and the protocol was approved by Sapienza University Research Committee (project identification code: RM118164220EB8B2).

### Analysis of adipose tissue miRNAs gene expression

An ABI PRISM 7500 Fast Real-Time PCR System (Life Technologies, Foster City, CA, USA) was used for the analysis process. Total RNA was isolated from paired surrounding adrenal neoplasia, peri-renal, and subcutaneous AT samples using TRIzol (Life Technologies, Grand Island, NY). cDNA was reversely transcribed from 5 ng total RNA samples using specific miRNA stem-loop primers from the QIAGEN^®^ RNeasy Lipid Tissue Mini Kit human panel. PCR products were synthesized from cDNA samples using sequence-specific primers from the QIAGEN^®^ miRCURY LNA RT Kit (QIAGEN GmbH, Hilden, Germany). We used hsa-miR-16 as endogenous controls to normalize the expression levels of miRNA target genes to correct the differences in the amount of cDNA loaded into PCRs (17). PCR was carried out by placing them 3 times on 96-well microtiter plates, and at the end of the reaction, the results were evaluated using ABI PRISM 7500 software. The cycle threshold value (Ct: cycle threshold) for each set of the three reactions was obtained by calculating the mean value.

### Statistical analysis

Data were expressed as mean and standard deviation (SD). Before statistical analysis, variables that showed a non-Gaussian distribution at the Kolmogorov–Smirnov test were transformed to achieve a normal distribution, and they were analyzed by non-parametric tests. Categorical variables were compared with the Fisher and chi-square tests. Continuous and categorical variables were compared at baseline and follow-up by the Mann–Whitney test. Relationships between continuous variables were assessed by calculating the Pearson correlation coefficient or the Spearman rank correlation coefficient when appropriate. Statistical analysis was performed using SPSS software (version 24 for Mac; IBM^®^, SPSS^®^Statistics, Italy). Significance was set at *p* < 0.05. The power of the study is 90.6% with a minimum sample size of 9 patients with beta 0.1 and alpha 0.05. The effect size is 1.61 (according to the Hedges' *g* formula).

## Results

Four men (36.4%) and 7 women (63.6%) affected by APA (with a mean age at the diagnosis of 43.86 ± 10.38 years old) and 2 men (40%) and 3 women (60%) affected by NFA (with a mean age at the diagnosis of 62.4 ± 4.5 years old) were evaluated before and after surgical tumor excision. Tables 1, 2 show clinical and biochemical parameters of enrolled patients affected by APA and NFA at baseline compared to follow-up from surgical treatment (mean follow-up: 12.1 ± 2.1 months). Surgical treatment was performed in all cases, and adrenal masses were adenomas on histological examination: 11 were APA and 5 were NFA. Patients with APA underwent laparoscopic adrenalectomy leading to complete remission of aldosterone excess based on the normalization of biochemical and clinical features at follow-up. At the first visit, the levels of plasma aldosterone concentration (PAC) and plasma renin activity (PRA) ratio were 71.97 ± 14.2 ng/ml:ng/ml/h vs. 6.8.1 ± 2.5 ng/ml:ng/ml/h after surgical treatment (*p* < 0.001). Notably, the office systolic and diastolic blood pressure (BP) values were significantly lower after treatment, with a significant reduction in the number of antihypertensive drugs (2.8 ± 1.2 vs. 1.0 ± 0.9, *p* = 0.001). Moreover, in patients with APA, we observed an improvement in body mass index (BMI) (28.3 ± 4.7 vs. 26.1 ± 3.18 kg/m^2^, *p* = 0.012), neck circumference (38.6 ± 5.2 vs. 37.71 ± 5.62 cm, *p* = 0.042), low-density lipoprotein (LDL) cholesterol (104 ± 33.2 vs. 94.86 ± 22.7 mg/dl, *p* < 0.001), triglycerides (92.8 ± 40.03 vs. 83.14 ± 26.6 mg/dl, *p* < 0.001), and uric acid (6.5 ± 2.13 vs. 5.55 ± 1.35 mg/dl, *p* = 0.02). On the other hand, we did not find significant differences between anthropometric and biochemical parameters at follow-up in patients with NFA. Table 3 summarizes data of 24-h ABPM parameters. After surgical treatment, 24-h systolic BP, diurnal systolic BP, diurnal diastolic BP, and nocturnal systolic BP were significantly decreased (133.80 ± 16.59 vs. 131.28 ± 17.18 mmHg, *p* = 0.046; 137.41 ± 16.15 vs. 133.75 ± 18.1 mmHg, *p* = 0.01; 86.04 ± 9.87 vs. 82.4 ± 8.9 mmHg, *p* = 0.013; 123.53 ± 19.00 vs. 120.1 ± 15.2 mmHg, *p* = 0.014, respectively). We did not find significant changes in 24-h ABPM parameters at follow-up in patients with NFA. Table 4 shows echocardiographic parameters. Remarkably, unlike subjects with NFA, patients with APA had an improvement in the left mass indexed for body surface area (LVMi) after surgery (102 ± 23.9 vs. 93.28 ± 23.04 g/m^2^, *p* = 0.034).

**Table 1:** Demographic and anthropometric data of all patients at baseline compared to follow-up.

		**Age (years)**	**Sex (M/F)**	**BMI (Kg/m** ^2^ **)**	**NC (cm)**	**WC (cm)**	**SBP (mmHg)**	**DBP (mmHg)**	**HR (bpm)**
APA (*n* = 11)	Baseline	43.8 ± 10.4	4/7	28.3 ± 4.7	38.6 ± 5.2	100 ± 14.2	152.8 ± 30.2	92.86 ± 19.11	71.43 ± 14.9
	Follow-up	45 ± 10.5	4/7	26.1 ± 3.2	37.7 ± 5.6	98 ± 19.3	135 ± 28.4	85.7 ± 14.8	70.4 ± 12.1
	*p*	0.001	NS	0.01	0.042	NS	<0.001	<0.001	NS
NFA (*n* = 5)	Baseline	62.4 ± 4.5	2/3	28.3 ± 2.6	36.7 ± 4.2	106.6 ± 7	138 ± 10.3	84 ± 5.5	67 ± 11.5
	Follow-up	63.4 ± 4.5	2/3	28 ± 1.6	36.1 ± 2.3	104.6 ± 1.2	135 ± 8.13	82 ± 3.25	65 ± 9.5
	*p*	0.001	NS	NS	NS	NS	NS	NS	NS

*APA, aldosterone-secreting adrenal adenoma; NFA, non-functioning adrenal mass; BMI, body mass index; NC, neck circumference; WC, waist circumference; SBP, systolic blood pressure; DBP, diastolic blood pressure; HR, heart rate*.

**Table 2:** Biochemical and hormonal data of all patients at baseline compared to follow-up.

		**Creatinine (mg/dl)**	**Glycaemia (mg/dl)**	**K^+^ (mEq/L)**	**Total cholesterol (mg/dl)**	**LDL cholesterol (mg/dl)**	**HDL cholesterol (mg/dl)**	**Triglycerides (mg/dl)**	**Uric acid (ml/dl)**	**PAC (pg/dl)**	**PRA (pg/ml)**	**AUR (μg/24 h)**	**UFC (nmol/24 h)**	**PC (nmol/l)**
APA (*n* = 11)	Baseline	1.03 ± 0.4	94.8 ± 12.1	4.2 ± 0.36	172.4 ± 32.6	104 ± 33.2	49.15 ± 11	92.8 ± 40	6.5 ± 2.1	295.1 ± 23.6	0.41 ± 0.2	19.9 ± 14.5	177.1 ± 98.2	341.8 ± 105.8
	Follow-Up	0.97 ± 0.3	94.5 ± 9.7	4.2 ± 0.2	165.5 ± 22.5	94.86 ± 22.7	50.86 ± 11.2	83.14 ± 26.6	5.55 ± 1.3	124.4 ± 73.2	1.8 ± 1.04	6.9 ± 6.1	167.3 ± 71.4	349.7 ± 114.9
	*p*	NS	NS	NS	NS	<0.001	NS	<0.001	0.02	<0.001	<0.001	<0.001	NS	NS
NFA (*n* = 5)	Baseline	0.95 ± 0.2	86.7 ± 11.7	4.5 ± 0.6	206.2 ± 25.6	115.8 ± 13	60.1 ± 13	87.7 ± 18.6	5.75 ± 0.8	88.2 ± 25.7	1.37 ± 0.3	10.5 ± 8.7	165.2 ± 77.8	306 ± 98.2
	Follow-Up	1.03 ± 0.3	88.3 ± 19.7	4.34 ± 0.9	207.3 ± 22.7	119.8 ± 11	56.1 ± 9.9	89.7 ± 11.6	5.95 ± 0.9	91.1 ± 27.3	1.47 ± 0.3	9.5 ± 2.7	170.2 ± 71.4	315 ± 68.1
	*p*	NS	NS	NS	NS	NS	NS	NS	NS	NS	NS	NS	NS	NS

*APA, aldosterone-secreting adrenal adenoma; NFA, non-functioning adrenal mass; K^+^, potassium; PAC, plasmatic aldosterone; PRA, plasmatica renin activity; AUR, 24-h urinary aldosterone; UFC (24-hurinary-free cortisol); PC, plasmatic cortisol*.

**Table 3:** Twenty-four-hour-ABPM data of all patients at baseline compared to follow-up.

		PAS-G (mmHg)	PAD-G (mmHg)	FC-G (bpm)	PAS-D (mmHg)	PAD-D (mmHg)	FC-D (bpm)	PAS-N (mmHg)	PAD-N (mmHg)	FC-N (mmHg)	DIPPING-S (Δ %)	DIPPING-D (Δ %)	DIPPING (%)
APA (*n* = 11)	Baseline	133.8 ± 16.6	82.5 ± 10.2	76.7 ± 9.1	137.4 ± 16.2	86 ± 9.8	78.5 ± 11.4	123.5 ± 19	72.2 ± 12.2	66.3 ± 8.6	8.9 ± 6.9	13.8 ± 8.3	45 ± 0.5
	Follow-up	131.3 ± 17.2	79 ± 7.7	75 ± 6.8	133.7 ± 18.1	82.4 ± 8.9	80.6 ± 7.2	120.1 ± 15.2	70 ± 6.4	65.6 ± 5.8	8.6 ± 4.79	12.3 ± 7.5	45 ± 0.5
	*p*	0.046	NS	NS	0.01	0.01	NS	0.01	ns	NS	NS	NS	NS
NFA (*n* = 5)	Baseline	125.3 ± 10.2	75.8 ± 4.8	70.5 ± 10.5	127.2 ± 7.88	73.2 ± 6.6	73.5 ± 4.5	122.12 ± 14.2	67.9 ± 4.89	64.7 ± 9.9	10.6 ± 7.24	11.8 ± 4.6	80 ± 0.4
	Follow-up	122.2 ± 8.5	74.8 ± 3.9	69.5 ± 9.2	125.2 ± 9.77	71.3 ± 5.6	71.6 ± 4.8	125.1 ± 17.2	69.9 ± 3.79	63.7 ± 7.9	9.6 ± 4.24	10.9 ± 4.5	60 ± 0.5
	*p*	NS	NS	NS	NS	NS	NS	NS	NS	NS	NS	NS	NS

*APA, aldosterone-secreting adrenal adenoma; NFA, non-functioning adrenal mass; PAS-G, 24-h systolic blood pressure; PAD-G, 24-h diastolic blood pressure; FC-G 24-h heart rate; PAS-D, diurnal systolic blood pressure; PAD-D, diurnal diastolic blood pressure; FC-D, diurnal heart rate; PAS-N, nocturnal systolic blood pressure; PAD-N, nocturnal diastolic blood pressure; FC-N, nocturnal heart rate; DIPPING-S, Systolic blood pressure dipping; DIPPING-D, Diastolic blood pressure dipping*.

**Table 4:** Echocardiographic data of all patients at baseline compared to follow-up.

		**LVEDD (mm)**	**IVSd (mm)**	**Aortic bulb (mm)**	**LAVI (m1/m2)**	**LVPWd (mm)**	**AO (mm)**	**LVMI (g/m** ^2^ **)**	**LVEF (%)**
APA (*n* = 11)	Baseline	49.5 ± 1.7	12.7 ± 4.9	33 ± 4.4	28 ± 3.3	9.3 ± 1.5	33.3 ± 4.5	102 ± 23.9	60.4 ± 3.6
	Follow-Up	49.3 ± 1.8	11.2 ± 4.1	32.4 ± 3.8	28.3 ± 2.6	9.3 ± 1.5	34 ± 3.6	93.3 ± 23	63.1 ± 2.4
	*p*	NS	0.042	NS	NS	NS	NS	0.034	NS
NFA (*n* = 5)	Baseline	48.6 ± 1.8	9.2 ± 1.2	34.2 ± 3.4	23.5 ± 4.5	9.25 ± 1.2	33.9 ± 1.6	75.2 ± 12.2	60.5 ± 3.7
	Follow-up	48.7 ± 1.8	9.5 ± 1.3	34.2 ± 3.4	24.6 ± 4.6	9.3 ± 1.6	34.9 ± 1.9	85.2 ± 12.8	60.9 ± 2.9
	*p*	NS	NS	NS	NS	NS	NS	NS	NS

*APA, aldosterone-secreting adrenal adenoma; NFA, non-functioning adrenal mass; LVEDD, left ventricular end-diastolic diameter; IVSd, end-diastolic interventricular septum; LAVI, left atrial volume indexed for body surface area; LVPWd, end-diastolic left ventricular posterior wall; LVMI, left ventricle mass indexed for body surface area; LVEF, left ventricular ejection fraction; AO, ascending aorta*.

### Expression of miRNAs in adipose tissue

Table 5 shows the quantitative expression of miRNAs (expressed in real-time PCR cycles) in the AT of patients with APA and NFA.

**Table 5:** Comparison between the real-time PCR cycle (Ct) averages for the different miRNAs analyzed, in the three types of AT of the patients with APA and NFA.

**APA = 11**	**Surrounding adrenal AT**	**Peri-Renal AT**	**Subcutaneous AT**	***p***
miR-132 (Ct)	35.67 ± 3.04°^*#^	32.25 ± 2.45^∧^	30.49 ± 0.77	^*^p < 0.05 vs. peri-renal AT • °*p* < 0.05 vs. subcutaneous AT
miR-143 (Ct)	29.56 ± 6.19°^#^	25.21 ± 5.39^∧^	22.75 ± 1.09	°*p* < 0.05 vs. subcutaneous AT
miR-221 (Ct)	30.83 ± 1.63°^#^	30.39 ± 1.7^°∧^	28 ± 1.18	°*p* < 0.05 vs. subcutaneous AT
miR-26a (Ct)	37.84 ± 0.44	37.11 ± 0.6	36.79 ± 1.71	NS
miR-26b (Ct)	37.44 ± 2.78°^#^	33.33 ± 0.96	32.49 ± 0.56	°*p* < 0.05 vs. subcutaneous AT
**NFA = 5**	**Surrounding adrenal neoplasia AT**	**Peri-Renal AT**	**Subcutaneous AT**	
miR-132 (Ct)	31.02 ± 2.42	29.42 ± 0.78	28.11 ± 0.35	NS
miR-143 (Ct)	22.35 ± 2.31	22.11 ± 0.47	21.10 ± 0.17	NS
miR-221 (Ct)	27.61 ± 0.78	27.62 ± 0.88	27.51 ± 0.64	NS
miR-26a (Ct)	34.91 ± 0.35	34.05 ± 0.81	35.06 ± 0.92	NS
miR-26b (Ct)	31.12 ± 1.52	32.24 ± 0.63	32.11 ± 0.35	NS
APA vs. NFA p	^#^*p* < 0.05 vs. NFA surrounding adrenal neoplasia AT	^∧^*p* < 0.05 vs. NFA peri-adrenal AT	NS	

*APA, aldosterone secreting adrenal adenoma; NFA, non-functioning adrenal mass; AT, adipose tissue*.

#### miRNA-132

Real-time PCR cycles were significantly higher in surrounding adrenal neoplasia AT of patients with APA than in peri-renal (35.67 ± 3.04 Ct vs. 32.25 ± 2.45 Ct, *p* < 0.05) and subcutaneous ATs (35.67 ± 3.04 Ct vs. 30.49 ± 0.77, *p* < 0.05). In patients with NFA, no significant differences were found in the comparison of real-time PCR cycles between the three types of AT analyzed. Real-time PCR cycles for miRNA-132 in fat surrounding adrenal neoplasia and in peri-adrenal AT were significantly higher in patients with APA than in patients with NFA (35.67 ± 3.04 Ct vs. 31.02 ± 2.42 Ct, *p* = 0.001 and 32.25 ± 2.45 Ct vs. 29.42 ± 0.78 Ct, *p* < 0.05, respectively).

#### miRNA-143

Patients with APA showed significantly increased levels of real-time PCR cycles for miRNA-143 in surrounding adrenal neoplasia AT compared to subcutaneous AT (29.56 ± 6.19 Ct vs. 22.75 ± 1.09 Ct, *p* < 0.05). No significant differences were found in the comparison of real-time PCR cycles between the three types of AT analyzed in patients with NFA. Real-time PCR cycles for miRNA-143 in fat surrounding adrenal neoplasia and in peri-adrenal AT were significantly higher in APA than in NFA (29.56 ± 6.19 Ct vs. 22.35 ± 2.31 Ct, *p* = 0.001 and 25.21 ± 5.39 Ct vs. 22.11 ± 0.47 Ct, *p* < 0.05).

#### miRNA-221

In the APA group, real-time PCR cycles were significantly higher in surrounding adrenal neoplasia AT and peri-renal AT compared to subcutaneous AT (30.83 ± 1.63 Ct vs. 28 ± 1.18 Ct, *p* < 0.05 and 30.39 Ct ± 1.7 vs. 28 ± 1.18 Ct, *p* < 0.05, respectively). No significant differences were found in the comparison of real-time PCR cycles between the three types of AT analyzed in patients with NFA. The comparison between fat surrounding adrenal neoplasia and peri-adrenal AT in APA and NFA shows higher real-time PCR cycle (Ct) averages in APA (30.83 ± 1.63 Ct vs. 27.61 ± 0.78 Ct, *p* < 0.5 and 30.39 ± 1.7 Ct vs. 27.62 ± 0.88 Ct, *p* < 0.05).

#### miRNA-26a

No changes were found in the comparison of real-time PCR cycles between the three types of AT analyzed in both groups.

#### miRNA-26b

Patients with APA showed significantly increased levels of real-time PCR cycles for miRNA-26b in surrounding adrenal neoplasia AT compared to subcutaneous AT (37.44 ± 2.78 Ct vs. 32.49 ± 0.56 Ct, *p* < 0.05). No significant differences were found in the comparison of real-time PCR cycles between the three types of AT analyzed in patients with NFA.

### Study of correlations

**Figure 1:**
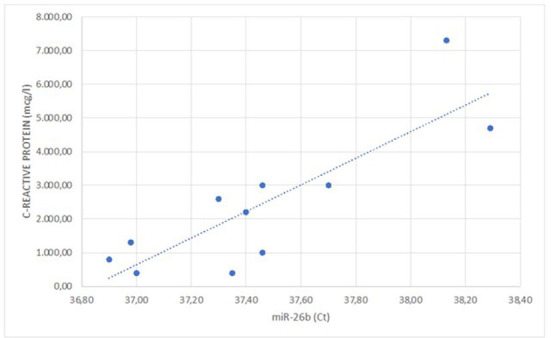
Linear correlation between the C-reactive protein values and the expression of miR-26b (*r* = 0.825; *p* = 0.043).

The correlation studies showed that plasma levels of C-reactive protein at baseline were positively correlated with real-time PCR cycles of miRNA-26b analyzed at surrounding adrenal neoplasia AT of the enrolled patients (*r* = 0.825; *p* = 0.043; Figure 1). Real-time PCR cycles for surrounding adrenal neoplasia AT miRNA-26b of enrolled patients also positively correlated with LVMi (*r* = 0.987; *p* = 0.002) and neck circumference (*r* = 0.818; *p* = 0.047).

**Figure 2:**
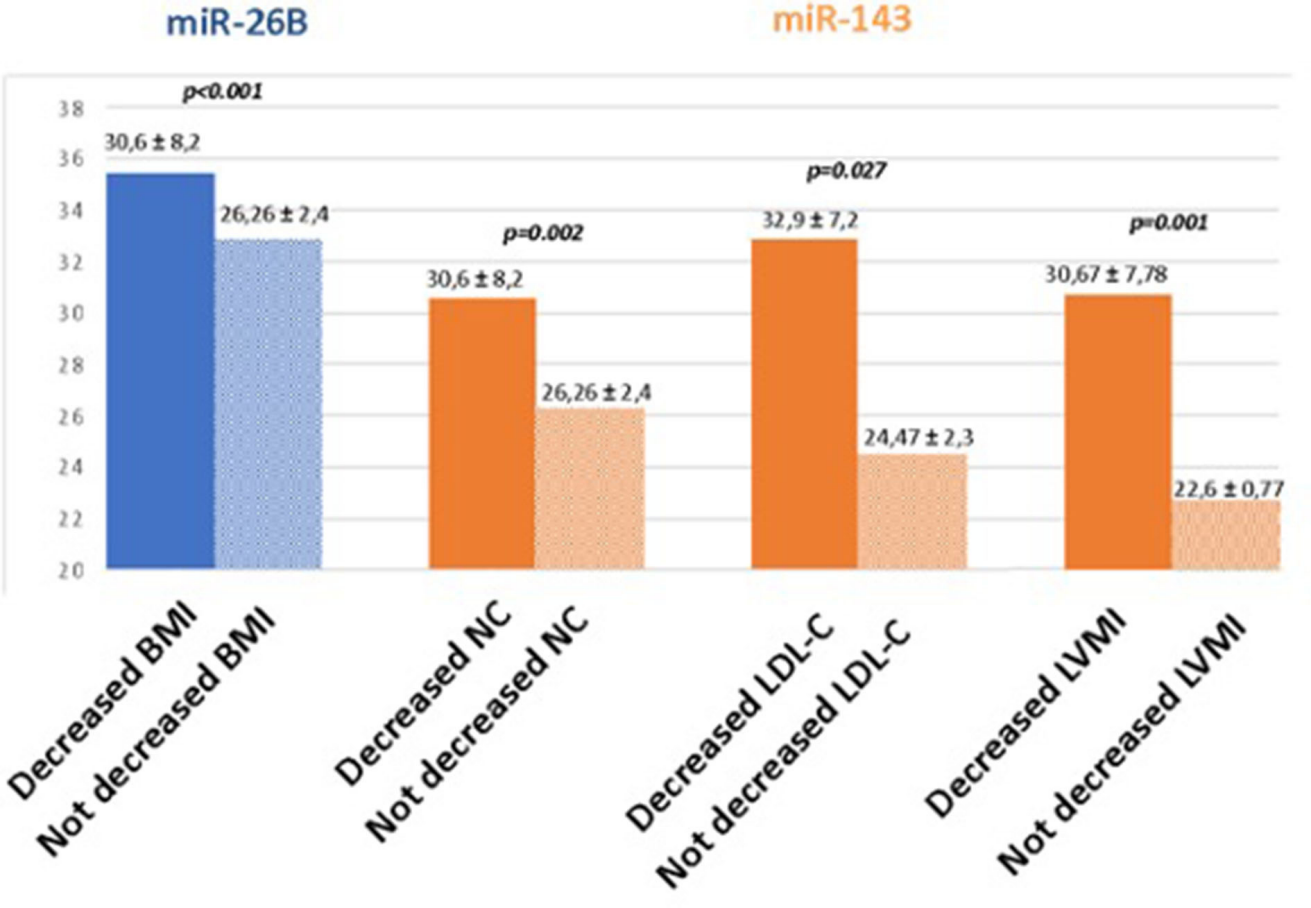
Comparison between the quantitative expression of surrounding adrenal neoplasia AT miRNAs and follow-up data. BMI, body mass index; NC, neck circumference; LDL-C, LDL cholesterol; LVMI, left ventricular mass indexed for the surface body area.

The comparison between the quantitative expression of surrounding adrenal neoplasia AT miRNAs and follow-up data is shown in Figure 2.

Patients with reduced BMI values at follow-up had significantly higher real-time PCR values of miR26b than those without improvement (35.5 ± 3.7 Ct vs. 32.9 ± 0.99 Ct, *p* < 0.001).

Furthermore, patients with an improvement in neck circumference and LDL cholesterol values at follow-up were those with reduced expression of miR-143 at surrounding adrenal neoplasia AT at baseline (30.6 ± 8.2 Ct vs. 26.26 ± 2.4 Ct, *p* < 0.002 and 32.9 ± 7.2 Ct vs. 24.47 ± 2.32 Ct, *p* < 0.027, respectively). Finally, patients with reduced LVMi had significantly higher real-time PCR values of miR-143 than those with no improvement (30.67 ± 7.78 Ct vs. 22.6 ± 0.77 Ct, *p* < 0.01).

## Discussion

Arterial hypertension is a multifactorial condition, associated with complex and different changes in BP and metabolic homeostasis; several studies showed different and interesting results in identifying specific miRNAs useful for differentiating patients with hypertension compared to normotensive subjects and in identifying the likelihood of developing arterial hypertension over time. Ye et al. (25) provided interesting data, albeit on a small population of a specific geographic area, evaluating some miRNAs in the Asian population, in patients with hypertension and normotensive subjects, finding an increased expression of some miRNAs (miRNA-198 and miRNA-1183) and a downregulation of others (miRNA-144-3p and miRNA-30e-5p) in subjects with hypertension. Similar results were found by Tao et al. (26), with the finding that patients with EH showed lower expression of miRNA-145-5p than the control group, especially in patients with obesity and patients who smoke.

Moreover, several miRNAs were evaluated with respect to the possible role not only in the development of arterial hypertension but also regarding hypertension-related organ damage. Mandraffino et al. (27) showed that, in EH, there are higher levels of two miRNAs (miRNA-221 and miRNA-222) involved in vascular remodeling and neo-angiogenesis processes, related to greater cardiac remodeling, and left ventricular hypertrophy; these miRNAs were also correlated with the inflammatory response depending upon excessive Ang-II and with increased production of oxygen-free radicals (ROS). Several authors highlighted relations between the altered expression of miRNAs and hypertension-related organ damage: upregulation of miRNA-29, miRNA-9, and miRNA-126 showed significant positive correlations with BP and some echocardiography parameters such as LVMI (28, 29); higher levels of miRNA-122 were positively correlated with myocardial remodeling markers (B-type brain natriuretic peptide, homocysteine, cardiac troponin T, and creatine kinase MB isoenzyme) and negatively correlated with myocardial remodeling (ejection fraction and left ventricular isovolumic relaxation time) (30); upregulation of microRNA-506-3p was correlated to the severity of hypertension, favoring vascular endothelial damage throughout increased cells apoptosis (31).

Chu et al. (32) found that the microRNA-136 expression was significantly increased in patients with EH, strictly correlating to all biochemical markers of RAAS system (ACE, renin, Ang II, and aldosterone), and those patients with higher behaviors of this miRNA showed better response to pharmacological treatment with calcium-antagonist. On the other hand, in patients with EH, He et al. (33) found downregulation of miRNA-483-3p and miRNA-27a-3p compared to normotensive subjects, inversely correlated to RAAS markers. Regarding the pathogenesis of RH, Karaa et al. (34) found higher levels of miRNA-21 in RH compared to EH and was positively correlated with aldosterone, age, office SBP, and 24-h ABPM all-day SBP; moreover, the AUC of miRNA-21 showed 83% of capacity to predict RH, with 95% sensitivity and 71% specificity. In an interesting study, Peng et al. (35) evaluated the effects of altered expression of miRNA-203 on the aldosterone production and tumorigenesis of adrenal cells; *in vitro* and in human models, these authors observed reduced expression in human APA samples than in peritumoral adrenal samples, inversely correlated with both plasma aldosterone level and tumor size; moreover, miR-203 inhibitors increased aldosterone production and cell proliferation in human adrenocortical cell line (HAC15); and in the mice model, selective inhibition of miR-203 led to increased systolic BP and plasma aldosterone levels. These authors found that mRNA-203 was able to influence aldosterone expression by activating the Wnt5a/b–catenin pathway. Similarly, mRNA-24 was found reduced in the tissue of patients with APA compared to normal adrenal tissue. This mRNA can interfere with cortisol and aldosterone syntheses binding on the untranslated region of CYP11B1 (11β-hydroxylase) and CYP11B2 (aldosterone synthase) genes (36). Previously, Carvajal et al. (37) evaluated in PA several mediators and novel biomarkers of chronic inflammation; in a finding that compared normotensive subjects and patients with EH, PA showed higher serum alpha-1-acid glycoprotein-1 (AGP-1) protein [specifically related to aldosterone, renin levels, and aldosterone-to-renin ratio (ARR)] and decreased the expression of miR-21-5p. AGP-1 could be evaluated with a dual role, as a potential biomarker of PA and as a possible mediator of the tissue response to high aldosterone production. The downregulation of miR-21-5p could affect the downstream target genes related to inflammation, interleukin-1β (IL-1 β) gene, and fibrotic processes (37). A study by Nakamura et al. (38) demonstrated that CYP11B1, a steroidogenic enzyme, which converts deoxycorticosterone to corticosterone, was diffusely detected in the APA, and it could play a pivotal role in the tumor genesis. In another recent study, Ahn et al. (39) showed lower CYP11B1 expression in adrenal adenomas of patients with PA compared with those affected by adrenal Cushing's syndrome. These findings underlined how the variable expression of steroidogenic enzymes in adrenal lesions is associated with the clinical heterogeneity of hormone overproduction.

To date, miRNAs have been demonstrated to play a major role in a wide range of developmental processes including cell proliferation, apoptosis, developmental timing, metabolism and immunity activation. Thus, a potential role of miRNAs was found in experimental studies on COVID-19 RNA-based drugs (40) and COVID-19 cerebrovascular events (41). Moreover, some miRNAs may represent potential targets for the treatment of acute myeloid leukemia (42) or in atherosclerosis for drug discovery (43). An interesting role in impaired insulin secretion in T2D pathogenesis was also found (44).

Primary aldosteronism is a frequent form of secondary hypertension, with a significant increase in cardiovascular mortality and morbidity, a high frequency of myocardial and vascular remodeling, a high frequency of arrhythmias (such as atrial fibrillation), and metabolic complications; therefore, the diagnosis and specific treatment of PA is fundamental for the reduction of the aforementioned complications (1).

In the literature, there are several articles, which aim to evaluate the usefulness of miRNAs as markers to differentiate EH from secondary forms of arterial hypertension. Decmann et al. (45) evaluated the circulating miRNA expression profiling in a large group of patients with PA, concluding that three analyzed miRNAs (miR-30e-5p, miR-30d-5p, and miR-7-5p) were significantly overexpressed in hyperplasia samples compared to adenoma samples. However, the evaluation of the ROC curves showed a poor diagnostic performance of these miRNAs in distinguishing hyperplasia from adenoma (sensitivity 58.7–61.7% and specificity 58.7–61.7%) when compared to adrenal vein sampling (sensitivity 92.5% and specificity of 100%). He et al. evaluated the possible effects of miRNAs regarding molecular and genetic aspects of PA, especially on adrenocortical cell proliferation and aldosterone production, found in patients with APA and compared to patients with unilateral adrenal hyperplasia and normal adrenal cortex, and significantly increased the expression of miRNa-26b and miRNA-26c and reduced the expression of miRNA-7 and miRNA-375 (inversely correlated with the tumor size). miRNA-375 exerts its tumor-suppressive function by reducing the expression of metadherin (MTDH) and phosphorylation and cell viability depending on Akt-Ser473, regulating cell proliferation and viability (46). The reproducibility of studies to identify biomarker-circulating miRNAs has proved difficult for other conditions due to a series of technical challenges (47).

In addition to the development of moderate–severe hypertension and RH, PA is associated with important target organ damage and the development of several complications related to pressure overload and to pleiotropic effects of aldosterone on the CV system; moreover, PA is characterized by several metabolic changes, such as metabolic syndrome (40), cardiac fibrosis (48), myocardial hypertrophy (4, 49), arrhythmias, HF, and deleterious effects on vascular remodeling, such as endothelial dysfunction, and oxidative stress, perivascular inflammatory lesions, fibrosis, platelets activation, and renal function (3, 5–7).

Regarding the pathophysiological mechanisms that can influence the development of metabolic alterations observed in patients with PA, we previously highlighted how altered balance between some important adipocytokines (an increase of leptin and resistin; and reduction of adiponectin) was related to the increased prevalence of metabolic syndrome and important cardiac remodeling (increased LVM, left atrium, and left ventricular end-diastolic volume; and reduction of ejection fraction) (3, 50).

This altered expression of adipokines was observed regarding the circulating levels of these molecules and the tissue expression in AT surrounding adrenal glands.

**Figure 3:**
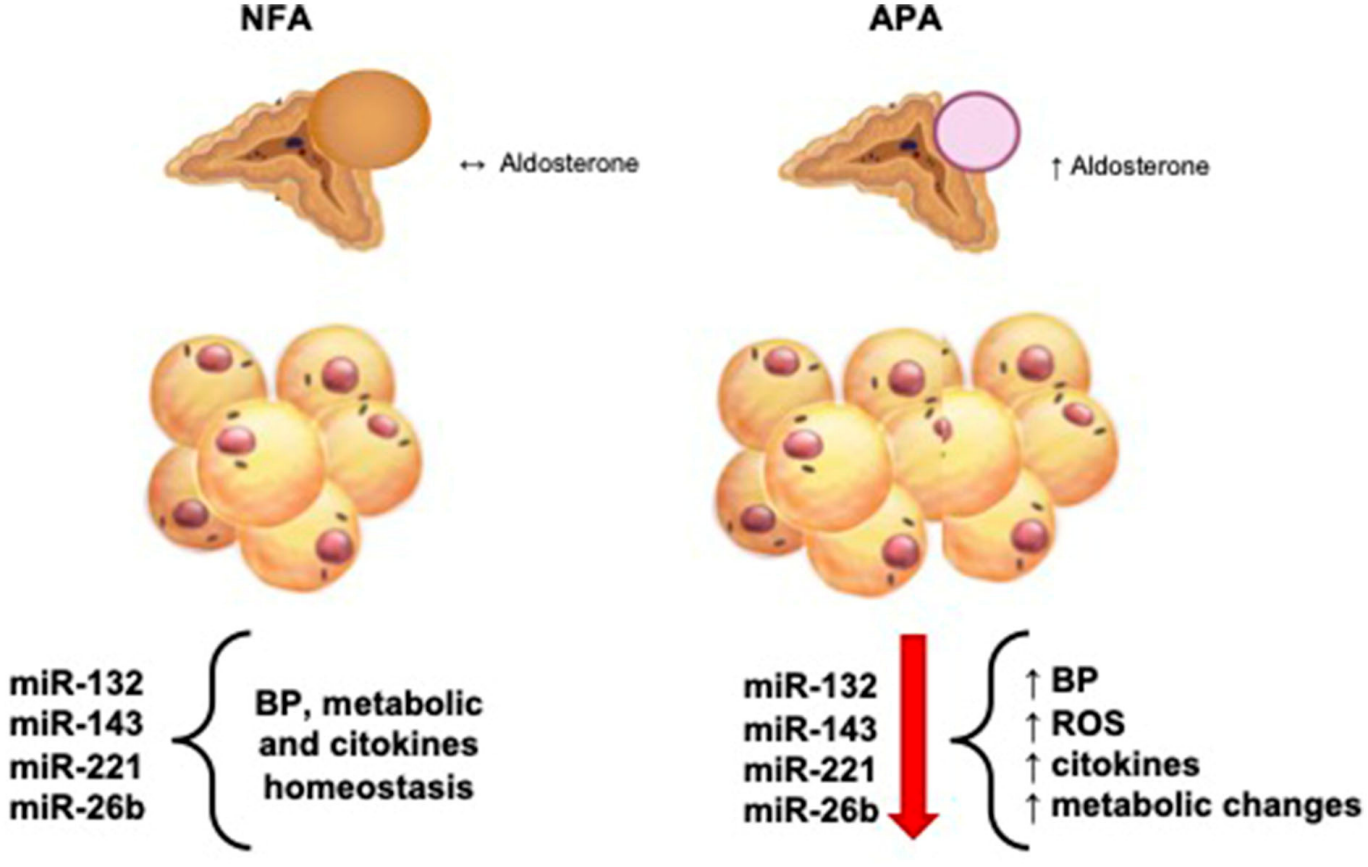
Physiological and pathophysiological relationship between aldosterone production and hemodynamic, metabolic, and molecular balance. NFA, non-functional adrenal mass; APA, aldosterone-secreting adrenal adenoma; BP, blood pressure; ROS, reactive oxigene species.

To date, there are no studies correlating altered expression of miRNAs in patients with PA, regarding the metabolic alterations observed in these patients. Interestingly, we found a significant reduction in the tissue expression of some miRNAs (miRNA-132, miRNA-143, and miRNA-26b) in visceral AT (peri-adrenal), whereas we did not observe alterations in subcutaneous AT, recognized as a deposit of AT without metabolic activity. Figure 3 summarizes the representation of the possible physiopathological relationships between the AT surrounding the adrenal gland in patients with APA and NFA and the analyzed miRNAs.

### miRNA-132

Previously, Heneghan et al. (10) showed a significant correlation of the tissue expression of miRNAs with respective circulating levels in several diseases, suggesting a possible role of such markers as non-invasive biomarkers for obesity and related diseases, and possible therapeutic targets. Among different miRNAs evaluated, these authors found a significant reduction of miRNA-132 expression in individuals with obesity compared to individuals without obesity, which is inversely related to BMI. Giardina et al. (51) evaluated that the variation of body composition is associated with different expression of some miRNAs, rather than with the use of different diet plans (low or high glycemic index and low-fat content), highlighting that in overweight and obese subjects, there was a significant relationship between variations of waist circumference and fat mass with the reduced tissue expression of miRNA-132.

Previously, Klöting et al. (17) evaluated the different tissue expression of some miRNAs between subcutaneous and omental fat deposits, highlighting a greater metabolic activity in the latter; these results were confirmed in our study, where we also observed, for some miRNAs, the different tissue expression in the visceral AT compared to the subcutaneous site, confirming a different metabolic role in these two districts.

The miRNA-132 is known to act in numerous ways in the regulation of complex metabolic pathways: through cAMP response element-binding protein, which regulates glucose homeostasis (52), and through the brain-derived neurotrophic factor, which is involved in the regulation of appetite and energy homeostasis (53). On the other hand, factors such as chronic hyperglycemia, altered cytokine profile, obesity, and systemic inflammation can alter the tissue expression of miRNAs, especially miRNA-132, playing a role in the link between AT dysfunctions and metabolic diseases such as type 2 diabetes mellitus (17).

Interestingly, miRNAs may have different effects depending on the tissue or organ evaluated; at the myocardial level, miRNA-132 appears to have different properties, especially in relation to the assessment of risk stratification of patients with HF. In recent controlled randomized studies conducted in chronic ischemic or hypertensive HF, the miR-132 inhibitor was assessed as a safe drug, determining a decrease of pro-BNP, significant narrowing of the QRS complex (54), reduced cardiomyocyte volume and interstitial fibrosis, improved capillary density, and increased left ventricular ejection fraction (55). At this level, miRNA-132 could act by inhibiting the expression of nuclear factor erythroid-2-related factor 2 (Nrf2), a pleiotropic protein, physiologically regulating the expression of several antioxidant genes and other cytoprotective enzymes, playing a key role in the maintenance of the functional integrity of cardiomyocytes and cardiac fibroblasts, and preventing maladaptive cardiac remodeling and HF (55).

On the other hand, at the liver site, miRNA-132 showed a negative correlation with pathogenic factors inducing inflammation and insulin resistance-related molecular pathways, playing a key role in the development of non-alcoholic fatty liver disease (NAFLD) and non-alcoholic steatohepatitis (NASH). In patients with obesity, reduced levels of miRNA were correlated to higher behaviors of LDL and total cholesterol, high expression of inflammation molecules [IL-6 and tumor necrosis factor-α (TNF-α)], liver macrophage infiltration, high cellular expression of V-rel reticuloendotheliosis viral oncogene homolog A (RELA), and STEAP family member 4 (STEAP4/STAMP2) (23). Moreover, in an interesting study conducted on brown AT (BAT), physiologically involved in the control of energy homeostasis acting by dissipating energy to produce heat and inversely related to BMI, glucose level, and metabolic syndrome, Kariba et al. (56) found that BAT, induced by norepinephrine or cold exposure, beyond adipokines, can induce higher expression of miRNA-132, repressing hepatic Srebf1 expression, attenuating expression of lipogenic genes, and controlling the lipid metabolism in the liver.

### miRNA-143

Recently, Wang et al. (57) showed different expressions of some miRNAs in the AT in women with obesity (93% of miRNAs were downregulated, including miRNAs-143 family), with significant correlations with numerous biological functions and signaling pathways. Similar results were shown in subjects with insulin resistance (58), in obese subjects with evident metabolic alterations (59), in young subjects developing dyslipidemia in childhood obesity (60), and in murine models subjected to marked weight gain (61). In this regard, Takanabe et al. (62) demonstrated contrasting results, showing higher miRNAs values correlated with elevated body weight and mesenteric fat mass levels in mouse models subjected to a high-fat diet.

For the first time in the literature, we showed a significant reduction in the tissue expression of miRNA-143 in visceral AT in patients with PA, suggesting the pathophysiological role of this molecule in promoting metabolic disorders in patients with PA.

Physiologically, the miRNAs-143 family is highly abundant in AT (59, 63); recently, Xie et al. (64) showed a significant reduction of these miRNAs in response to an increased inflammatory level, in particular, secondary to elevated behaviors of TNF-α secreted by macrophages. Regarding oxidative stress, we previously found increased oxidative stress in patients with PA due to the direct effect of aldosterone on the production of NOX_2_, the catalytic subunit of the main supplier of oxidative stress (NADPH oxidase) (6).

Altered expression of miRNA-143 results in impaired processes of differentiation, maturation, and adipogenesis of white adipocytes (65), inducing inhibition of new adipocyte proliferation, favoring triglycerides accumulation into mature adipocytes, characterized by larger volume adipocytes (66), and reducing the expression of specific adipocyte genes (proliferator-activated peroxisome receptor-γ2 and glucose transporter-4) (67). In this respect, in human adipocytes, Zhu et al. (68) showed inhibition of miRNA-143 by higher plasma levels of free fatty acids, leptin, and resistin.

In this regard, we showed that significant metabolic alterations (hyperglycemia, dyslipidemia, and high rate of metabolic syndrome) observed in patients with PA were associated with increased expression of leptin and resistin in the visceral AT and consensual reduced expression of adiponectin, suggesting a relevant role of these adipokines in the development of metabolic changes observed in patients with PA (3). In this study, we found significantly reduced tissue expression of miRNA-143 in patients with APA compared to subjects with NFA; and in addition, patients with lower tissue expression of miRNA-143 (expressed by greater number of real-time PCR cycles) showed more clinical improvement to the specific treatment of excess hormone, characterized by more evident reduction of neck circumference and LDL behaviors; moreover, lower tissue expression of miRNA-143 was associated with a significant reduction of myocardial mass of left ventricle during follow-up after definitive treatment.

Recent studies suggest the protective role of miRNAs in the cardiovascular system; regarding the crosstalk between AT and the cardiovascular system, it has been shown that, under shear stress, endothelial cells release increased the levels of miR-143, internalized by the smooth muscle cells (SMCs) of the middle tunic in order to avoid SMC de-differentiation, exerting a protective role against atherosclerotic plaque (69); lower levels of this miRNA can favor cardiovascular remodeling observed in patients with PA.

The miRNA-143 could act through different pathways; miR-143 was first identified as a positive regulator of human adipocyte differentiation in 2004 *via* effects on ERK5 signaling (70). Moreover, further analysis showed that miRNA-143 family overexpression or inhibition, respectively, decreased or increased the MAPK7, suggesting that MAPK7 is a target gene of miR-143-3p mediating cell proliferation and differentiation; inhibition of MAPK7 reduces adipocyte proliferation, playing an opposite role in pre-adipocyte proliferation and differentiation when compared with the miRNA-143 (71). Recent studies showed that HMGA2, a target gene of some miRNAs produced in adipocytes, which is a transcription factor that can regulate adipogenesis (60, 70, 72, 73), is upregulated in subjects with obesity and patients with diabetes (74) and correlated with increased body weight and body fat (75), whereas HMGA2 knockout mice have less fat, low-fat content, and are not susceptible to obesity-related diseases (76). Wang et al. showed that the overexpression of HMGA2 is related to the reduction of miRNA-143, accompanied by a greater inflammatory state in ATs (57), which is particularly associated with the activation of macrophages, with the production of pro-inflammatory cytokines, including TNF-α, IL-6, and IL-1β (77, 78).

### miRNA-26b

We found a significant reduction of miRNA-26b expression on the AT surrounding the adrenal gland in patients with APA compared to the NFA group. In an interesting study, Wróblewski et al. (79) demonstrated that chronic or transient hyperglycemia induces changes in the expression of several adipokines, characterized by lower behaviors of adiponectin and higher production of IL-6 in human visceral preadipocytes, suggesting some mechanisms of development of high grade of inflammation in patients with diabetes. Regarding this, in addition to higher IL-6 expression, multiple miRNAs (in particular miRNA-26b family) were found to be downregulated in chronic hyperglycemia. In this respect, we found a significant negative relation between miRNA-26b expression and the circulating levels of C-reactive protein (expressed as a correlation between high real-time PCR cycles of miRNA-26b and elevated values of C-reactive protein: *r* = 0.825, *p* < 0.05). Strum et al. showed that TNF-α, leptin, and resistin induced downregulation of the hsa-miR-26b expression in adipocytes, the latter acting as an important mediator in regulating the obesity-related insulin sensitivity and inflammatory responses (21). Several studies reported a reduction of plasma miRNA-26 family in patients with diabetes (80, 81), and the overexpression of miRNA-26 in murine model protects against high-fat diet-induced obesity, whereas miR-26 deficiency results in AT hyperplasia (82).

Moreover, the expression level of miR-26b negatively correlates with increasing BMI and homeostasis model assessment for IR in human obese subjects. BMI is widely known as an independent risk factor for developing HF, CAD, stroke, and overall CVD death, and the reduction of BMI is related to the improvement of the metabolic and BP profile in these patients. We have demonstrated that patients with higher expression of miRNA-26b showed greater benefit by definitive treatment of aldosterone excess, evidenced by a greater reduction in BMI during follow-up after surgical treatment. miR-26b could promote insulin-dependent glucose uptake through glucose transporter type 4 translocation to the plasma membrane in human mature adipocytes. miR-26b modulates insulin-stimulated AKT activation *via* inhibition of its target gene, PTEN, and significantly increases insulin sensitivity *via* the PTEN/PI3K/AKT pathway (83). Recently, Ma et al. showed that miR-26b is highly abundance in AT, acting as an effective regulator of adipogenesis; overexpression of miR-26b increased expression of adipogenic marker genes and lipid accumulation in pre-adipocyte, suggesting its regulation on pre-adipocyte differentiation. miRNA-26b could act through inhibition of expression of fibroblast growth factor 21 (FGF21) mRNA, important growth factor in regulating glucose and lipid metabolism (84), or repressing expression of Fbxl19, encoding a component of SCF–type E3 ubiquitin ligase complexes (82).

### Limitations

The limitations of our study are the reduced number of cases studied; moreover, we did not evaluate the circulating levels of miRNAs, requiring to evaluate tissue expression of miRNAs, to verify different tissue expressions of miRNAs; in fact, in literature, there is a high variability of the results due to different methodologies used, and the evaluation of organs of different dimensions can induce insignificant changes in circulating miRNA levels, albeit a significantly modified tissue expression. Therefore, our study aimed to evaluate the tissue expression of miRNAs precisely to avoid possible methodological biases, and further studies will be necessary to evaluate the possible correlation between tissue expressivity and circulating levels of miRNA, validating the plasma dosage (85).

## Conclusions

In conclusion, in the last decades, several studies highlighted the role of different miRNAs in favoring different diseases (various types of cancers, metabolic diseases, and cardiovascular diseases); regarding the diagnostic setting of arterial hypertension, some miRNAs have been studied in the stratification of the risk of arterial hypertension, development of moderate–severe or resistant arterial hypertension, and metabolic complications. This study, conducted on tissue expression of miRNAs, highlights, for the first time, the possible pathophysiological role of some miRNAs in determining the metabolic and vascular alterations in patients with PA. Further studies are needed to correlate the tissue expression of these miRNAs to biohumoral markers and instrumental data on increased cardiovascular risks.

## Data availability statement

The raw data supporting the conclusions of this article will be made available by the authors, without undue reservation.

## Ethics statement

The studies involving human participants were reviewed and approved by Sapienza University of Rome. The patients/participants provided their written informed consent to participate in this study. Written informed consent was obtained from the individual(s) for the publication of any potentially identifiable images or data included in this article.

## Author contributions

LP and AC wrote the main manuscript text, tables, and figures. MB, GD, GC, and FCip provided the resources. DD conducted the investigation. FCir and PR worked on data curation. LP, AC, and CL worked on the conceptualization of the study. FCip, MS, and CL provided the supervision. All authors reviewed the manuscript.

## Funding

This study was supported by Sapienza University of Rome funding (RM118164220EB8B2).

## Conflict of interest

The authors declare that the research was conducted in the absence of any commercial or financial relationships that could be construed as a potential conflict of interest.

## Publisher's note

All claims expressed in this article are solely those of the authors and do not necessarily represent those of their affiliated organizations, or those of the publisher, the editors and the reviewers. Any product that may be evaluated in this article, or claim that may be made by its manufacturer, is not guaranteed or endorsed by the publisher.
